# Acute and chronic stress prevents responses to pain in zebrafish: evidence for stress-induced analgesia

**DOI:** 10.1242/jeb.224527

**Published:** 2020-07-22

**Authors:** Jack S. Thomson, Anthony G. Deakin, Andrew R. Cossins, Joseph W. Spencer, Iain S. Young, Lynne U. Sneddon

**Affiliations:** 1 School of Environmental Sciences, University of Liverpool, Liverpool L69 3GP, UK; 2 Department of Electrical Engineering and Electronics, University of Liverpool, Liverpool L69 3GJ, UK; 3 Institute of Integrative Biology, University of Liverpool, Liverpool L69 7ZB, UK

**Keywords:** *Danio rerio*, Animal welfare, Stress, Nociception, Endogenous analgesia, Pain

## Abstract

The state of an animal prior to the application of a noxious stimulus can have a profound effect on their nociceptive threshold and subsequent behaviour. In mammals, the presence of acute stress preceding a painful event can have an analgesic effect whereas the presence of chronic stress can result in hyperalgesia. While considerable research has been conducted on the ability of stress to modulate mammalian responses to pain, relatively little is known about fish. This is of particular concern given that zebrafish (*Danio rerio*) are an extensively used model organism subject to a wide array of invasive procedures where the level of stress prior to experimentation could pose a major confounding factor. This study, therefore, investigated the impact of both acute and chronic stress on the behaviour of zebrafish subjected to a potentially painful laboratory procedure, the fin clip. In stress-free individuals, those subjected to the fin clip spent more time in the bottom of the tank, had reduced swimming speeds and less complex swimming trajectories; however, these behavioural changes were absent in fin-clipped fish that were first subject to either chronic or acute stress, suggesting the possibility of stress-induced analgesia (SIA). To test this, the opioid antagonist naloxone was administered to fish prior to the application of both the stress and fin-clip procedure. After naloxone, acutely stressed fin-clipped zebrafish exhibited the same behaviours as stress-free fin-clipped fish. This indicates the presence of SIA and the importance of opioid signalling in this mechanism. As stress reduced nociceptive responses in zebrafish, this demonstrates the potential for an endogenous analgesic system akin to the mammalian system. Future studies should delineate the neurobiological basis of stress-induced analgesia in fish.

## Abstract (precis)

**Summary:** Exposure of zebrafish to acute or chronic stress prior to fin clipping prevents behavioural changes normally seen after fin clip; naloxone treatment prevented this effect, demonstrating stress-induced analgesia.

## INTRODUCTION

The impact of stress on mammals prior to a noxious, tissue-damaging stimulus can have profound effects on the processing and behavioural expression of pain or nociception (Butler and Finn, 2009). Rather than being a fixed, unidirectional response, pain is bidirectional and can be subdued by descending modulatory pathways. Stress prior to a painful event can modulate these descending pathways and increase the nociceptive threshold of animals, allowing a form of analgesia that permits the performance of other behaviours such as anti-predatory behaviour (Amit and Galina, 1986); this process is known as stress-induced analgesia (SIA), which can act as a confounding factor in experimental studies (Butler and Finn, 2009; Sorge et al., 2014). A recent study highlighted the significance of this phenomenon when stress induced by human male but not female handlers resulted in significant reductions in the expression of standardized pain behaviour in rodents (Sorge et al., 2014). While SIA has been explored in detail in mammalian model organisms, only a few studies have explored this phenomenon in fish and have focused on the South American piauçu fish (*Leporinus macrocephalus*) (Alves et al., 2013; Wolkers et al., 2013; 2015a,b; 2017). The ability of SIA to modulate the nociceptive response of the model fish species zebrafish (*Danio rerio*), in response to common but potentially painful procedures, is currently unknown in adults.

In the laboratory environment, fish are often subjected to a host of acute stressors; for example, individuals held in air while being transferred between tanks during procedures, or moved from environments containing many conspecifics to being socially isolated in recovery tanks. White et al. (2017) demonstrated that holding zebrafish in groups enhanced their recovery from fin clipping compared with zebrafish held in isolation. If stressors are repeated often over time, this could also function as a chronic stressor which, if similar to that observed in other species, could have vastly different implications for how pain is modulated. In mammalian species, for example, chronic stress can induce long-lasting hyperalgesia with increased sensitivity to pain (Jennings et al., 2014) whereas acute stress can result in SIA with reduced responses to pain (Costa et al., 2005; Da Silva Torres et al., 2003).

In order for fish to experience SIA there needs to be appropriate neurochemical pathways to facilitate the modulation of the nociceptive response. In mammals, SIA is mediated by several different neurochemical systems, including opioid, µ-aminobutyric acid (GABA), glutamate, cannabinoid, serotonin, noradrenaline (norepinephrine) and stress hormones (Butler and Finn, 2009), which are all highly conserved in zebrafish (Gonzalez-Nunez and Rodriguez, 2009). In mammals, the amygdala is a pivotal brain region involved in the mediation of SIA; in fish, the telencephalon is thought to be functionally homologous to the mammalian amygdala (Broglio et al., 2005). A recent study in piauçu fish (*L. macrocephalus*) identified the importance of the GABAergic system within the telencephalon in mediating SIA (Wolkers et al., 2015b). Additionally the endo-cannabinoid system has also been implicated in SIA in piauçu, demonstrating that these mechanisms may be evolutionarily conserved (Wolkers et al., 2015a; 2017). As well as fish having similar neurochemical pathways, behavioural experiments have also provided evidence for the presence of SIA in fish. Rainbow trout (*Oncorhynchus mykiss*) subjected to stress appeared to show a diminished nociceptive response in relation to the application of a noxious stimulus (Ashley et al., 2009), while the piauçu fish, when exposed to a predator cue, also demonstrated a reduction in nociceptive behaviours (Wolkers et al., 2013). This response in piauçu fish was further inhibited by the injection of naloxone, an opioid antagonist, suggesting that SIA is strongly modulated by the endogenous opioid system. Stress-induced hyperalgesia (SIH) is less well understood, partly because of the complicated interplay between neurotransmitter systems and multiple brain regions. In rodents, a range of chronic stressors such as social defeat, forced swim tests and vibrational/noise stress have all resulted in an increased sensitivity to pain of thermal, mechanical and visceral origin (Jennings et al., 2014): this phenomenon is yet to be explored in fish.

Zebrafish are one of the most popular species of fish used in experimentation because of a suite of characteristics (short generation time, transparent embryos, detailed genomic information available, high homology with humans, etc.) that make them highly desirable as a model species for a wide range of scientific disciplines (Hill et al., 2005; Löhr and Hammerschmidt, 2011; McCluskey and Postlethwait, 2015). This broad adoption of zebrafish as a model species ensures that they are now subject to a diverse array of invasive procedures that may result in tissue damage. Empirical evidence indicates that fish fulfil the criteria for animal pain (Sneddon, 2015; Sneddon et al., 2014) as they possess a nociceptive system similar to that found in mammals (Ashley et al., 2006; 2007; Sneddon, 2003b; 2015; 2018; 2019; Sneddon et al., 2003) and express molecular and physiological changes during potentially painful stimulation in higher brain areas (Dunlop and Laming, 2005; Nordgreen et al., 2007; Reilly et al., 2009; 2008b). Observations have also revealed that fish perform abnormal behaviours in response to noxious events that can last for between 3 and 6 h, indicating a prolonged complex change in behaviour (Ashley et al., 2009; Millsopp and Laming, 2008; Reilly et al., 2008a; Roques et al., 2010; Sneddon, 2003a; White et al., 2017; Schroeder and Sneddon, 2017). A recent study in our laboratory demonstrated the potential for the fin clip, a commonly used laboratory procedure, to be painful in zebrafish (Deakin et al., 2019a,b; Schroeder and Sneddon, 2017; White et al., 2017). Compared with control and sham-handled fish, fin clipped individuals showed a significant reduction in movement complexity (possibly indicating stereotypical behaviour), a preference for the bottom of the tank as well as decreases in tank exploration and average speed of swimming (Deakin et al., 2019a,b; Thomson et al., 2019): this behavioural change was not transient and lasted for up to 6 h. Further, these behavioural responses to fin clipping were reduced by the use of pain-relieving drugs (Deakin et al., 2019a,b; Schroeder and Sneddon, 2017; Thomson et al., 2019).

The aim of this study was to address the impact of acute and chronic stress on the modulation of behavioural responses induced by a tail fin clip, a procedure routinely conducted for genomic screening (http://zfin.org/zf_info/zfbook/chapt7/7.8.html) and in fin amputation studies (e.g. Azevedo et al., 2011). This procedure has altered the behaviour of not only zebrafish (Deakin et al., 2019a,b; Thomson et al., 2019) but also Nile tilapia, *Oreochromis niloticus* (Roques et al., 2010). Based upon results from mammalian studies it was hypothesized that the application of acute stress would have an analgesic (SIA) effect, with fish showing reduced or no responses to fin clip. Conversely, the application of chronic stress may have a hyperalgesic (SIH) effect, causing an elevated response to fin clipping. The laboratory environment may involve potential stressors during husbandry and experimental treatment that, if not accounted for, could result in discrepancies between laboratories carrying out the same experiments; this has important implications for the refinement of zebrafish research, and the reproducibility and uniformity of procedures (Gerlai, 2019). To test the phenomenon of SIA, the ability of naloxone to ameliorate the effects of SIA was assessed by administering naloxone to block the endogenous opioid system to counteract the effects of stress on behavioural responses. To our knowledge, this is the first study to address the impact of chronic stress and endogenous analgesia in fish.

## MATERIALS AND METHODS

### Subjects and husbandry

This research received local ethics approval from the University of Liverpool and was conducted under UK Home Office Guidelines (PPL 40/3534). Eight month old female zebrafish [*Danio rerio* (F. Hamilton 1822)] of AB strain (*n*=54; mean±s.e.m. size 0.85±0.09 g) were randomly selected from one clutch (reducing genetic variability) from the University of Liverpool aquarium in-house breeding project for experiments; only females were used to prevent the confounding effect of sex in the stress response (e.g. Rambo et al., 2017); however, it is known that males and females do not differ in their behaviour in response to painful treatment (Costa et al., 2019a; Taylor et al., 2017). Females are conspicuous by their large abdomen compared with the torpedo shape of males and sex was confirmed at the conclusion of the experiments by examining the ovaries. Stock fish were housed in a semi-closed recirculation system in 10 l tanks with constant aeration at 27±1°C on a 14 h:10 h light:dark cycle. Fish were randomly selected then netted carefully for transfer from the stock tanks into a semi-closed recirculation system in a 3 l tank, consisting of two parallel rows of glass tanks (20×30×20 cm; *n*=1 fish per tank). Each tank was fitted with an identical, external laminated printout of a green plant background; this allowed the easy detection of the focal fish by an in-house tracking system because of the enhanced contrast provided by the green background as described in Deakin et al. (2019a). All tanks were supplied with filtered fresh water (pH 7.2, NH_3_ ≤0.01 mg l^−1^, NO_2_ ≤0.01 mg l^−1^, NO_3_ ≤5 mg l^−1^) maintained on the same temperature and light regime as above. Aeration was provided by an aerated, 200 l biological filter with one-third of the water replaced weekly. Fish were acclimatized in their experimental tank for 2 weeks prior to experimentation and fed twice daily *ad libitum* with a commercial tropical ornamental flake (TetraMin). All fish used in experiments had fed when food was presented for at least 7 days prior to experimentation. Fish were in chemical (through shared water) and visual contact with adjacent tanks so had social contact until the evening prior to experimentation, when two opaque pieces of plastic were placed between tanks to visually isolate the test individuals. Before the experiment commenced, the flow to the test tanks was turned off to prevent chemical communication between subjects. At the end of all experiments, the zebrafish were killed humanely (concussion and pithing) and the brain tissue collected for another study.

### Treatment groups

**Fig. 1.**
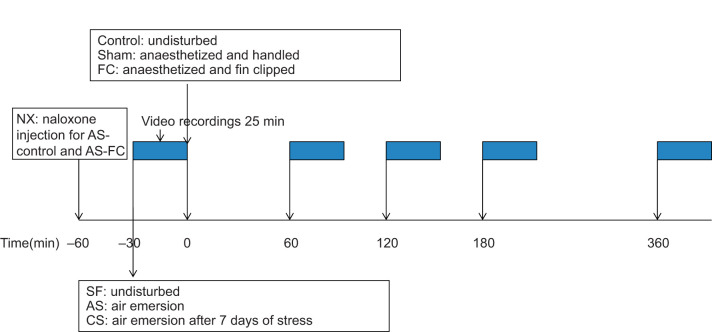
**Diagrammatic schedule of the experimental protocol.** Zebrafish were undisturbed and stress free (SF), acutely stressed (AS) or chronically stressed (CS), where fish in both AS and CS groups were subject to 1 min of air emersion prior to the first video recording. At 0 min, fish were assigned to control, sham (anaesthetized and handled) or fin-clipped (underwent tail fin clipping under anaesthesia; FC) groups. Some individuals were also administered with naloxone (NX) at 30 min prior to air emersion before being assigned to the control or FC groups (*n*=7 per group). Video recordings (25 min duration) were made at the times indicated on the diagram.

This study sought to investigate the ability of stress to modulate the nociceptive response of zebrafish to a previously described painful procedure, the fin clip. The fin clip was chosen as previous studies have demonstrated that there are profound changes in behaviour that are ameliorated by the use of analgesic drugs (Deakin et al., 2019a,b; Schroeder and Sneddon, 2017; Thomson et al., 2019). Fish were randomly assigned to the groups (*n*=7 per group calculated using power analysis as the minimum effective sample size to meet the study's objectives) described in Table 1: stress-free (SF) individuals were not stressed prior to treatment; acute stress (AS) groups had air emersion imposed prior to fin clipping; and chronic stress (CS) groups had stressors applied for 7 days prior to treatment. To investigate SIA, a further two groups were tested: acute stress with naloxone and fin clip (NX-FC) and acute stress with naloxone but no fin clip (NX-control) to investigate the effects of naloxone alone without fin clip. Naloxone was not used in the chronic stress treatment for reasons outlined below. The experimental schedule is detailed in Fig. 1. Treatments were conducted in a random order to prevent any sequence effects, except for the naloxone groups, which were conducted last after the experimental data from the acute and chronic stress groups had been analysed. Tests were randomized within the naloxone groups. Fish within the fin clip groups were carefully netted and transferred to a 1 l beaker containing 500 ml of aerated water dosed with benzocaine (0.033 g l^−1^; Sigma-Aldrich Co.) to anaesthetize fish. Benzocaine was used as it has short-lasting analgesic properties and fish were taken to deep plane anaesthesia where they were unconscious during the noxious treatments, as shown by a lack of reflex responses (Sneddon, 2011). Fin-clipped fish had 40% of their caudal fin carefully removed as described in the Zebrafish Handbook (http://zfin.org/zf_info/zfbook/chapt7/7.8.html) before being returned to their home tank and allowed to recover from the anaesthesia. Control fish in the AS, CS and NX treatments were anaesthetized and handled in the same manner but did not receive a fin clip.

**Table 1.**
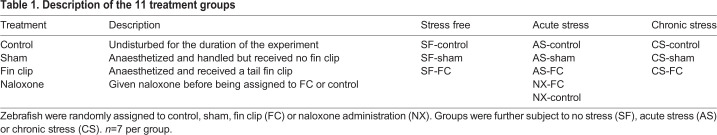
**Description of the 11 treatment groups**

### Acute stress

On the morning of the experiment (approximately 09:00 h GMT), fish (*n*=14) were carefully netted out of their home tank and subjected to a standard acute stressor, air emersion, for 1 min (Ramsay et al., 2009); care was taken to prevent other fish from observing this procedure and that the focal fish was secure in the net. Fish were then returned to their tank and monitored to ensure they showed no overt response to the stressor; none did. After the air emersion, the first video was recorded to observe baseline behaviour (Fig. 1). After this video had been recorded, fish were either anaesthetized and subjected to the fin clip procedure (*n*=7) or anaesthetized but not subjected to fin clip (*n*=7) before videos were recorded at 60, 120, 180 and 360 min after this procedure (Fig. 1).

### Chronic stress

For 1 week prior to the day of the intervention during the 14 day acclimation period, fish (*n*=7) were exposed daily to one of three stressors: 1 min air emersion (as described above), 1 min chasing the individual around their tank with a net or 1 min confinement, where fish were netted and gently held against the side of the tank while still submerged in water; a random combination of these stressors carried out at different times of the day was adopted to prevent habituation. These stressors were chosen based on their effectiveness in previous studies (Piato et al., 2011; Yue et al., 2004). During exposure to these stressors, opaque plastic was placed between adjacent tanks to prevent other fish observing the treatment. The pre-treatment stressor was applied at 09:00 h GMT and the first pre-intervention video was recorded immediately afterwards. Fish were then undisturbed (CS-control), anaesthetized but no fin clip applied (CS-sham) or subjected to the fin clip procedure (CS-FC; *n*=7 per group) and behaviour was recorded at 60, 120, 180 and 360 min after the procedure (Fig. 1).

### Impact of naloxone on SIA

Preliminary data analysis showed that acutely stressed fish (AS-FC) exhibited possible evidence of SIA with reduced responses to the fin clip compared with the SF-FC group (see Results). Further, acute and chronic stress alone (without fin clip) did not significantly affect behaviour compared with that of SF-control undisturbed fish (see below for details). Therefore, we only tested naloxone in the acute stress group. To test the SIA hypothesis, fish were randomly assigned to one of two groups NX-FC (*n*=7) or NX-control (*n*=7; Table 1). At approximately 08:30 h GMT, fish were anaesthetized as described above and injected intraperitoneally with naloxone (30 mg kg^−1^) using a sterile gastight syringe and needle (34 g; Hamilton, Bonaduz, Switzerland) following the procedure of Wolkers et al. (2013), which allows the naloxone time to diffuse. This procedure took less than 1 min and fish were returned to their home tank to recover. After 30 min, fish were then subjected to the acute stress (1 min air emersion) and the first video was recorded; half of the fish received the fin clip as outlined above. Videos were then recorded at 60, 120, 180 and 360 min time points (Fig. 1).

### Data collection

Fish behaviour was recorded prior to fin clip (after the stressor) then at intervals afterwards; each recording lasted 25 min (Fig. 1). The first recording ensured that the impact of stress on normal behaviour was measured. Fish were tracked using two industrial IDS USB 3.0 colour video cameras (IDS, Obersulm, Germany) fitted with a 25 mm monofocal lens and connected to a computer (HP compact elite 8300, Palo Alto, CA, USA) running tracking software developed at the University of Liverpool (Alzu'bi, 2015). Cameras positioned above and to the side of the focal tank were used to track the 3D trajectories of fish. Cameras positioned above the tanks were mounted on a sliding gantry of 1.4 m length, enabling cameras to be moved between tanks with minimal disturbance. Cameras positioned to the side of the tanks were attached to tripods 1.4 m away from the focal tanks and were manually moved between tanks; the movement of cameras always occurred the night before an experiment to minimize disturbance during the day of the experiment. Data files generated by the 3D tracking software were then processed with software in MATLAB (version 2014; Deakin et al., 2019a) to calculate the average swimming speed (cm s^−1^). Using this software, it was also possible to divide the tank in half horizontally and calculate what percentage of each 25 min video recording zebrafish spent in the bottom half of the tank (% bottom time). Fractal dimension analysis was also applied to each video as described in Deakin et al. (2019b). The fractal dimension is a measure of complexity (Mandelbrot, 1967), derived from the 3D movement and swimming coordinates of the focal fish, that produces a single value, the fractal dimension; a more complex swimming pattern was identified by higher fractal dimension values and a more repetitive, less complex pattern was identified by lower fractal dimension values, which has been shown to reflect poor welfare (Deakin et al., 2019b). These three parameters (swimming speed, % bottom time and fractal dimension) are known to be the most important behaviours affected by tail fin clipping from previous studies, where fractal dimension and swimming speed were profoundly reduced and the use of the bottom of the tank increased (Deakin et al., 2019a,b; Schroeder and Sneddon, 2017; Thomson et al., 2019). All videos were analysed blind and the video identity only revealed once data collection was complete.

### Statistical analysis

Effects of stress, treatment and time were assessed for each of swimming speed, time spent at the bottom of the tank and fractal dimension by using linear mixed models (lme4; Bates et al., 2015). In each case, explanatory variables and the full interaction term were included in the initial model, and the effect of time was coded as a quadratic function based on *a priori* expectations of the responses (Deakin et al., 2019a,b). Stress (SF, CS, AS) and treatment (control, sham, FC) were considered as categorical fixed effects, time as a continuous covariate, and subject was included as a random effect. To determine the significance of interaction terms or their component main effects, where relevant, terms were step-wise removed from the model and models compared using a log-likelihood approach. Terms were removed where comparison against the more complete model was not significantly different (α=0.05); where models did significantly differ, the model with the lower Akaike information criterion (AIC) was selected. Assumptions were assessed visually using probability and residuals versus fits plots. Speed was log_10_-transformed to account for heteroscedasticity in the raw data, and there was evidence of a ceiling effect in time spent at the bottom.

To determine the effects of naloxone on fish behaviour and responses to fin-clipping, an identical procedure was followed. For appropriate analysis, however, only a subset of the full range of treatment groups was incorporated into the full model: three treatment groups without naloxone (1: SF-control; 2: SF-FC; 3: AS-FC) and two with naloxone (4: acute stress control, NX-control; and 5: acute stress fin-clip, NX-FC). For modelling, no full interaction of an effect of stress and naloxone was available and so these factors were combined into a single factor, stress-naloxone, with three levels: no stress, acute stress, acute stress plus naloxone. The full model included one second-order interaction (treatment×stress-naloxone×time) and three first-order interactions (stress-naloxone×time, stress-naloxone×treatment, treatment×time). To meet assumptions, speed did not need to be log_10_-transformed; however, bottom time (%) was logit-transformed; fractal dimension did not require transformation.

All analyses were conducted in R (http://www.R-project.org/). The results presented below focus on the effects of fin-clipping on fish behaviour, the impact of stress on these effects, and how such impacts are influenced by naloxone. Where other trends in the data were observed, analyses are presented without interpretation.

## RESULTS

**Fig. 2.**
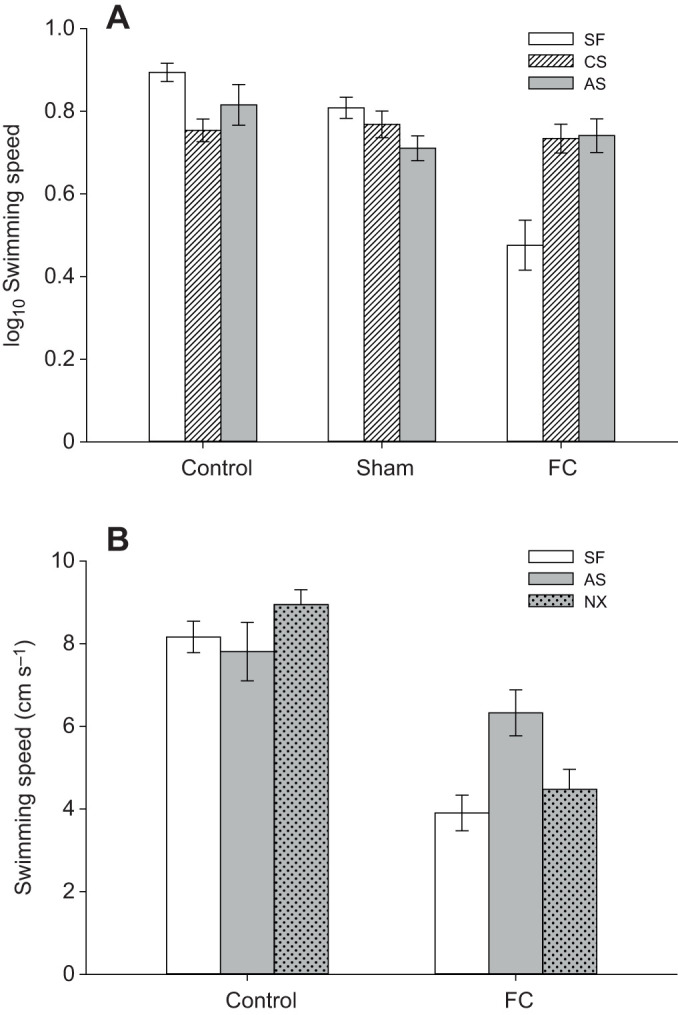
**Speed of swimming in zebrafish.** (A) Mean (±s.e.m.) log_10_ speed (cm s^−1^) of control, sham or tail fin-clipped (FC) zebrafish (*n*=63). Fish were additionally stress free (SF), or either chronically (CS) or acutely stressed (AS). (B) Mean (±s.e.m.) speed of SF, AS and acutely stressed fish treated with naloxone (NX) zebrafish (*n*=7 per group) when held in control conditions or FC.

**Fig. 3.**
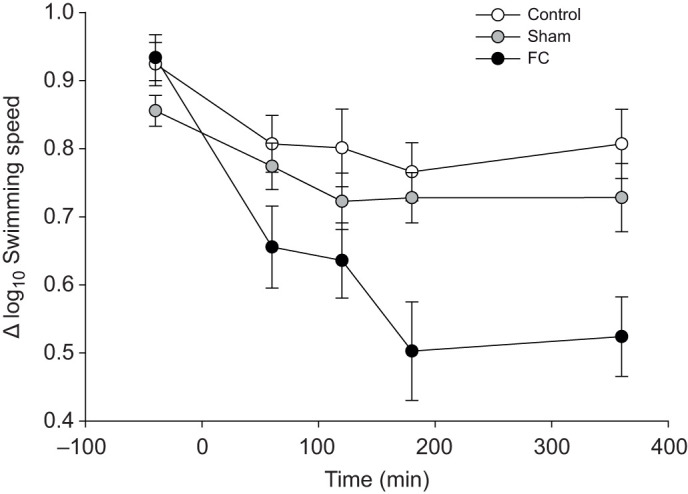
**Impact of fin clipping on swimming speed in zebrafish.** Change in mean (±s.e.m.) log_10_ speed (cm s^−1^) of all control, sham or tail fin-clipped (FC) zebrafish groups (*n*=21 per treatment) over a 6 h period.

Stress had a minimal effect on the behaviour of control groups (AS-control and CS-control), AS-sham and CS-sham zebrafish. Fin-clipped fish were on average the slowest moving fish, particularly when unstressed (SF-FC); stressed fish (AS-FC, CS-FC) swam at a speed closer to that of control and sham fish regardless of the type of stress (Fig. 2A). This increase of speed in AS-FC and CS-FC fish after stress appeared to be lost when zebrafish were treated with naloxone, as their speed was no different from that of SF-FC fish (Fig. 2B; Fig. S1). Regardless of stress, FC fish slowed the most over time and did not appear to recover (Fig. 3). The full interaction was marginally non-significant for fish speed (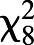
=15.27, *P=*0.054); however, speed was significantly influenced by two-way interactions of stress×treatment (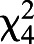
=16.379, *P*=0.003) and time×treatment (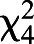
=33.18, *P*<0.0005). The two-way interaction of stress×time was not significant (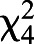
=1.54, *P*=0.819). When examining the effect of naloxone, the three-way interaction of treatment×stress-naloxone×time was marginally non-significant (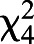
=8.48, *P*=0.076), but there was a significant interaction of treatment×stress-naloxone (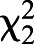
=6.50, *P*=0.039). There was also a significant interaction of treatment×time (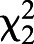
=27.37, *P*<0.0005) but not of time×stress-naloxone (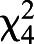
=0.95, *P*=0.917).

**Fig. 4.**
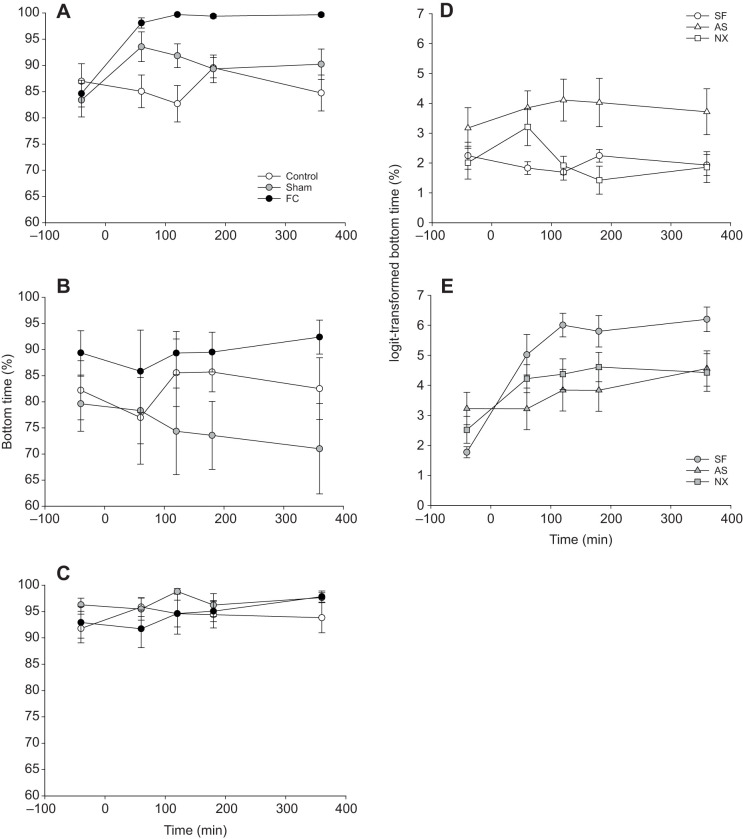
**Use of tank bottom by zebrafish.** (A–C) Mean (±s.e.m.) percentage of time zebrafish spent at the bottom of the tank when (A) stress free (SF) or exposed to (B) chronic stress (CS) or (C) acute stress (AS) (*n*=63). Fish were also maintained as control, sham (grey) or tail fin clipped (FC). (D,E) Control (D) and FC (E) zebrafish (*n*=7 per group) were also SF or AS, or exposed to acute stress and naloxone (NX).

In general, SF-FC and CS-FC fish spent the most time at the bottom of the tank compared with control and sham fish (Fig. 4A,B), although this difference was absent in AS fish (Fig. 4C). SF-sham and SF-control fish behaved similarly when under no stress. Under chronic stress, all fish spent less time at the bottom of the tank, with CS-sham fish spending the least amount of time at the bottom (Fig. 4B). NX-control fish spent approximately the same duration of time as SF-control zebrafish at the bottom of the tank, which was less than AS-control fish (Fig. 4D). When fish were fin clipped, NX prevented the behavioural change induced by stress that was seen in SF-FC fish, with NX-FC bottom time similar to that of AS-FC fish (Fig. 4E). The full three-way interaction was a significantly better fit than a reduced model for time spent at the bottom of the tank (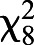
=16.96, *P*=0.030; Fig. 4A–C). The full three-way interaction was also a significantly better fit than a reduced model when examining the effect of naloxone (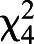
=26.46, *P*<0.0005; Fig. 4D,E).

**Fig. 5.**
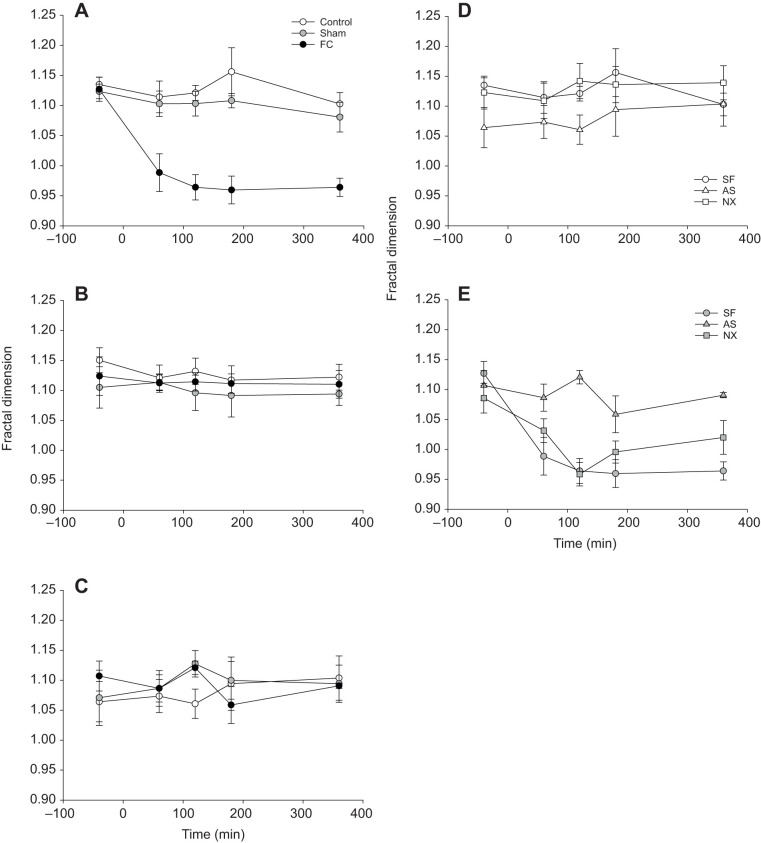
**Fractal dimension analysis of zebrafish swimming trajectories.** (A–C) Mean (±s.e.m.) fractal dimension for zebrafish (*n*=21 per treatment) when (A) stress free (SF), or exposed to (B) chronic stress (CS) or (C) acute stress (AS). Fish were also maintained as control, sham or tail fin clipped (FC). (D,E) Control (D) and FC (E) zebrafish (*n*=42) were also SF or AS, or exposed to acute stress and naloxone (NX).

SF-FC fish exhibited a decrease in fractal dimension over time compared with SF-control and SF-sham fish and did not recover (Fig. 5A). Under CS, the decrease in fractal dimension for FC fish was lost (Fig. 5B). Under AS, while there were few differences in fractal dimension between AS-control, AS-sham and AS-FC fish, fractal dimension overall was lower (Fig. 5C), suggesting AS reduced the complexity of swimming. NX-control and NX-FC fish appeared to behave similarly to SF-control and SF-FC fish both in control conditions (where fractal dimension for both was generally higher than for AS-control fish; Fig. 5D) and when FC (where fractal dimension for both was generally lower than for AS-FC fish; Fig. 5E). The full three-way interaction was a significantly better fit for fractal dimension than a reduced model (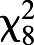
=30.01, *P*<0.0005; Fig. 5A–C). Likewise, the full three-way interaction was a significantly better fit than a reduced model when considering the role of naloxone (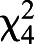
=11.04, *P*=0.026; Fig. 5D,E).

## DISCUSSION

The purpose of this study was to investigate whether the behavioural response provoked by the fin clip procedure could be modulated by the prior experience of either acute or chronic stress and to investigate the presence of either stress-induced analgesia (SIA) or stress-induced hyperalgesia (SIH). The fin clip procedure elicited a substantial change in behaviour where stress-free female zebrafish (SF-FC) exhibited less complex swimming patterns (reduced fractal dimension), a reduction in activity, identified by slower average swimming speeds, and increased use of the bottom of the tank as has been recorded in other studies (Deakin et al., 2019a,b; Schroeder and Sneddon, 2017; Thomson et al., 2019; White et al., 2017). This was not due to a mechanical change in tail length as administering pain-relieving drugs restores normal behaviour in fin-clipped zebrafish (Deakin et al., 2019a,b; Schroeder and Sneddon, 2017). In the present study, both AS and CS had an anti-nociceptive or analgesic effect on FC zebrafish, resulting in behaviours that did not differ significantly from those of stress-free undisturbed controls (SF-control). These findings are similar to those observed in previous studies using fish models (Alves et al., 2013; Wolkers et al., 2013; Maximino, 2011) and provide more evidence for the phenomenon of SIA in fish. The use of naloxone confirmed the responses were indeed SIA in AS fish as NX-FC zebrafish showed similar behaviour to SF-FC fish in average speed and fractal dimension but to a lesser extent in time spent at the bottom. No evidence of SIH was found in the CS-FC group as these zebrafish did not exhibit enhanced responses to the fin clip, such as reductions in swimming speed, lower fractal dimension scores or increased use of the bottom of the tank. Surprisingly, neither acute nor chronic stress had significant effects upon normal behaviour compared with control or sham groups that did not receive a fin clip either before or during the experiment.

In mammals, SIA is a component of the flight or fight mechanism and is activated in stressful situations and/or where there is potential for injury. The presence of this phenomenon across several species of fish (Alves et al., 2013; Wolkers et al., 2013; Maximino, 2011) highlights its evolutionary importance as it is seemingly well conserved across the vertebrate phylum (Butler and Finn, 2009). Stress seemed to subdue the behavioural response to fin damage; however, this effect lasted up to 360 min after fin clipping. Studies profiling the stress response in zebrafish show cortisol, a stress hormone, returns to baseline levels at 3 h post-stressor (Cortés et al., 2018); therefore, the effects of stress in the present study may still have an influence after 3 h. The provision of an acute stressor prior to the injection of formaldehyde in the piauçu fish similarly led to a reduction in nociceptive behaviour, albeit for a shorter period of 10 min (Alves et al., 2013). The differing SIA response of piauçu and zebrafish is probably due to the species-specific differences in pain-related behaviours that exist (e.g. Reilly et al., 2008a); the response to pain in piauçu, for example, involves an increase in activity as opposed to that observed in zebrafish, which typically involves a reduction in activity (Deakin et al., 2019a; Correia et al., 2011; Lopez-Luna et al. 2017a,b,c,d; Magalhães et al., 2018; Reilly et al., 2008a; Schroeder and Sneddon, 2017; Taylor et al., 2017; Thomson et al., 2019).

The results from the present study demonstrate that a painful treatment affects behaviour profoundly and that there is evidence for the descending control of pain through SIA. In mammals, the rostroventromedial medulla (RVM) and periaqueductal gray (PAG) are particularly important (Dafny, 1997; Heinricher et al., 2009). The RVM can inhibit nociceptive information and is key in the control of descending pain processes whereas the PAG gets inputs from several brain regions and can provide an analgesic effect. These two areas have a combined influence on responses to pain and nociception as input from cortical and subcortical areas allows the PAG and RVM to diminish the intensity of pain. This can be replicated experimentally via administration of opioids (prevented by naloxone) or electrical stimulation in rats (Reynolds, 1969) and humans (Hosobuchi et al., 1977; Richardson and Akil, 1977a,b; Tsou and Jang, 1964). Fish also possess these brain areas and thus it is likely that these are candidates for descending control of pain (Wullimann, 1998; Rink and Wullimann, 2004; Kittelberger et al., 2006). Future studies are required to investigate the role of the PAG and the RVM in central pain mechanisms in zebrafish and other fish species after acute and chronic stress.

The administration of either acute or chronic stress alone did not result in any clear behavioural changes in AS-control or CS-control zebrafish. Air emersion is a standard stressor in fish (e.g. rainbow trout; Pounder et al., 2016) and decreased swimming behaviour was observed in the AS treatment where all groups (control, sham and FC) spent much longer at the bottom than SF and CS groups. This would suggest the air emersion was indeed stressful. However, SIA was observed in the AS-FC and CS-FC groups in terms of average speed and fractal dimension; thus, it would seem that the CS treatment reflected an acute stressor rather than chronic stress. Studies by Piato et al. (2011) and Rambo et al. (2017) found that chronic stress led to the activation of the physiological stress axis as well as reductions in activity and an increase in time spent in the bottom of the tank after 14 days of unpredictable stress. In these studies, after a 7 day period, as used in the present study, there were behavioural differences and whole-body cortisol elevation, but Piato et al. (2011) found no difference in activity at this time point. Further, these studies used a larger number of stressors (two randomly applied per day for 7–14 days) such as crowding, cooling, warming and lowered water levels that were not logistically possible in our study. Instead, we chose only three stressors from these studies and applied these randomly 3 times per day over an 8 h period in the hope that this would not allow the fish to recover homeostasis over this period. It may be that either these 1 min stressors were simply not that stressful or they were not applied for a long enough duration: Piato et al. (2011) applied 8 min of net chasing whilst Ghisleni et al. (2012) and Rey et al. (2015) both applied 15–90 min of confinement stress. This may mean the zebrafish in the present study did not find the three stressors stressful enough and so habituated to them, perceiving them to be non-threatening. Zebrafish habituate to being moved to a novel tank after 3 days when transferred daily, so habituation occurs relatively quickly (Gaspary et al., 2018). Furthermore, most of the studies employing chronic unpredictable stress paradigms held fish in groups and used social isolation as a stressor. A recent study has demonstrated that zebrafish recover more slowly when held individually as opposed to group-housed fish, which appear more resilient to stressors (White et al., 2017), and this may explain the discrepancies between the studies. Piato et al. (2011) explored the impact of chronic stress on groups of males, yet sex differences in stress responses have been recorded in zebrafish (Oswald et al., 2012; Cortés et al., 2018) and to prevent any sex effects the present study used females only, as males and females do not differ in their response to painful treatment (Costa et al., 2019a; Taylor et al., 2017). Social grouping and sex can also have significant impacts on the behavioural responses to stress in mammals (Beery and Kaufer, 2015). Keeping social animals singularly, particularly in a barren environment, can be a source of anxiety and act as a chronic stressor in zebrafish (Collymore et al., 2015). It is, therefore, possible that our SF-control group may have also been stressed to some degree, which may explain the absence of a significant difference between stressed and non-stressed individuals. However, our animals were feeding for at least 7 days prior to experimentation and anorexia is a primary response to stress in fish (Cortés et al., 2018), making this explanation unlikely in the present study. It would be interesting to test this theory with the administration of drugs such as diazepam, which is known to be anxiolytic (Bencan et al., 2009; Egan et al., 2009; Lopez-Luna et al., 2017d), or etomidate, which suppresses cortisol (Lopez-Luna et al., 2017d), in the control groups to see whether that enables a clearer distinction between stressed (AS-control and CS-control) and non-stressed (SF-control) individuals. Indeed, in larval zebrafish, diazepam and etomidate suppress the behavioural responses to anxiety and stressful treatments, respectively (Lopez-Luna et al., 2017d). Further, future studies aimed at investigating SIH and the impacts of chronic stress on pain in zebrafish should employ a more severe protocol than we have used here. Of course the alternative explanation is that SIH does not exist in fish but given the similarities between the teleost and mammalian mechanisms of nociception and pain (Sneddon, 2015; 2018; 2019), this needs to be investigated. The impact of adopting an unpredictable chronic stress paradigm on inhibitory avoidance learning in zebrafish resulted in the expression of stress genes and an elevation in cortisol, suggesting the effectiveness of the protocol at inducing stress (Manuel et al., 2014). However, in the same study, the ability to learn to avoid an electric shock was impaired, suggesting that the pain of the shock was not severe or, we propose, that SIA was in play; this led the authors to suggest that in this instance chronic SIH was unlikely (Manuel et al., 2014).

Koolhaas et al. (2011) make an important point about how to define stress and that animals can display an increase in glucocorticoids through hypothalamic–pituitary–adrenal/interrenal (HPA/I) axis activity in response to events that increase arousal such as feeding, rewarding situations and intra-specific interactions. These authors state stress should only be applied when the challenge or stressor is unpredictable or uncontrollable and disrupts homeostasis. Certainly in the present study, our stressful treatments in both the AS and CS groups were uncontrollable. However, in the CS paradigm it may be that zebrafish habituated or learned that the randomly applied stressors were not life-threatening and as such there was an element of predictability or control. Further, previous studies have shown that the responses to pain can be influenced by social interactions. For example, dominant rainbow trout returned to a familiar hierarchy after painful treatment reduced aggressive behaviour towards known hierarchy members but when returned to an unfamiliar hierarchy, these individuals were more aggressive and sought to establish their dominance (Ashley et al., 2009). The authors concluded that establishing dominance took priority over exhibiting signs of pain. An alternative explanation is that SIA came into play and actually reduced pain, allowing the dominant individual to behave more aggressively. Additionally, zebrafish subject to painful treatment recover much more quickly in a group than when held in isolation or in dominant–subordinate pairs (White et al., 2017). It may be that the social interactions within a group increased arousal and the HPI, inducing SIA; thereby, animals appeared to recover more quickly. However, this would require further testing using naloxone to fully understand whether SIA is an explanation for these results.

SIA is typically explored through the administration of the opioid antagonist naloxone. In mammals, acute stress is known to activate neurochemical pathways that involve the binding of endogenous opioids to opioid receptors (Akil et al., 1986; Amit and Galina, 1986), a process that can be blocked by naloxone (Butler and Finn, 2009). Zebrafish have a highly comparable stress response to mammals (Löhr and Hammerschmidt, 2011), with stress eliciting the release of endorphins that act as ‘natural’ pain-relieving substances in the nervous system through binding to opioid receptors and inhibiting nociception and pain. In this study, the injection of naloxone, an opioid receptor antagonist, prevented the anti-nociceptive effect of AS, resulting in behaviours that were closer to those observed in the SF-FC group. Average swimming speed and fractal dimension were similar in the NX-FC to those in the SF-FC group. This effect of naloxone was seen to a lesser extent in time spent at the bottom, with intermediate values between SF-control and SF-FC. Fractal dimension reduces the 3D complex swimming trajectories of zebrafish to one value and has been applied to a variety of painful treatments in zebrafish to produce an arbitrary scale of pain intensity (Deakin et al., 2019b). Any fractal dimension values above 1.08 are reflective of normal healthy zebrafish; below this value, individuals can be classified as acutely stressed (1.03–1.08), or experiencing pain (mild, 0.97–1.03; moderate, 0.94–0.97; and severe (<0.94). The values obtained from the SF-FC group certainly reflect moderate to severe pain for the duration of the experiment. The CS-FC and AS-FC groups were within the normal to stressed fractal dimension range. The fractal dimension from the NX-FC group indicates stress through to mild and moderate pain so it would seem naloxone does have an effect on fractal dimension, raising the values from those associated with moderate–severe pain. When exploring the data, naloxone appears to increase swimming speed of NX-FC fish at 360 min (Fig. S1) and reduce time spent at the bottom at 180 min. This suggests naloxone may influence behaviour in complex ways and this may mean NX-treated individuals were relatively more active, resulting in a high fractal dimension in NX-FC after 180 min. Alternatively, the effects of naloxone may be wearing off towards the end of the experiment. Previous studies have only explored the effects of naloxone for up to 60 min after administration in zebrafish (e.g. Bosse and Peterson, 2017; Costa et al., 2019b); thus, future studies should explore the duration of action in fish. In mammals, naloxone has a short duration of action (∼15–30 min), requiring re-administration in cases of opioid overdose (Gerak et al., 2019). Naloxone is also known to affect behaviour in rodents, with mice being more anxious and displaying hyperalgesia (Bertolini et al., 1978; Grevert and Goldstein, 1977), although in the present study naloxone did not significantly affect swimming speed, fractal dimension or time spent at the bottom of the tank in NX-control zebrafish. Future studies should explore this phenomenon in more detail, experimenting with other agonists and antagonists. In mammals, SIA is governed by a complex interplay of neurotransmitters/neuropeptides including GABA, glycine, vasopressin, oxytocin, adenosine, endogenous opioids and endocannabinoids (Butler and Finn, 2009). As well as endogenous opioids (Wolkers et al., 2013), both GABA and endocannabinoids have recently been shown to play a role in SIA in fish (Wolkers et al., 2015a,b; 2017), suggesting that the SIA response in zebrafish is influenced by both GABA and cannabinoid antagonists and these studies have shown that a key brain area for the modulation of pain and SIA is the dorsomedial telencephalon, which is homologous to the mammalian amygdala (Wolkers et al., 2017). Evidence of SIA has been found in zebrafish larvae at 5 days post-fertilization, where a stressful experience prior to exposure to a potentially painful stimulus prevented the associated reduced activity (Lopez-Luna et al., 2017d). This was confirmed by our findings in adult zebrafish.

### Conclusion

Fin clipping, a painful treatment, results in behavioural changes in adult female zebrafish of AB strain. Future studies should explore the responses of males and other genetic strains to determine whether these responses are widespread. It would be important to test wild-caught zebrafish or their offspring to understand the ecological significance of SIA. Both CS and AS had a SIA effect on FC zebrafish and prevented the reduction in swimming speed, reduced fractal dimension score and increased use of the lower half of the tank. Thus, stress may provide a confounding factor on studies of pain in zebrafish (Sneddon, 2017) and this should be considered in future experimentation by allowing fish to recover from stress prior to data collection. Eliminating stress may enhance reproducibility and intra-specific variation and represents an important refinement in the use of zebrafish. The SIA effect was prevented via the prior administration of the opioid antagonist naloxone, indicating the importance of the endogenous opioid system in modulating the pain response in zebrafish. This study presents evidence for the descending control of pain in a non-mammalian vertebrate, demonstrating that this phenomenon is evolutionarily conserved. Although there was no evidence of SIH, further research exploring more intense stressors is needed to fully determine its presence and to untangle the modulation of pain in this zebrafish. These results highlight the potential for prior experience to modulate the response of zebrafish to a laboratory procedure and this may present a confounding effect that has implications for both biomedical and biological research that uses zebrafish as a model species.

## Supplementary Material

Supplementary information
