# Supplementary material for: Acute and chronic stress prevents responses to pain in zebrafish: evidence for stress-induced analgesia
Source: J Exp Biol. 2020 Jul 22;223(14):jeb224527. doi: 10.1242/jeb.224527 (PMC7391404; doi:10.1242/jeb.224527)
Supplement: Supplementary information [file jexbio-223-224527-s1.pdf]

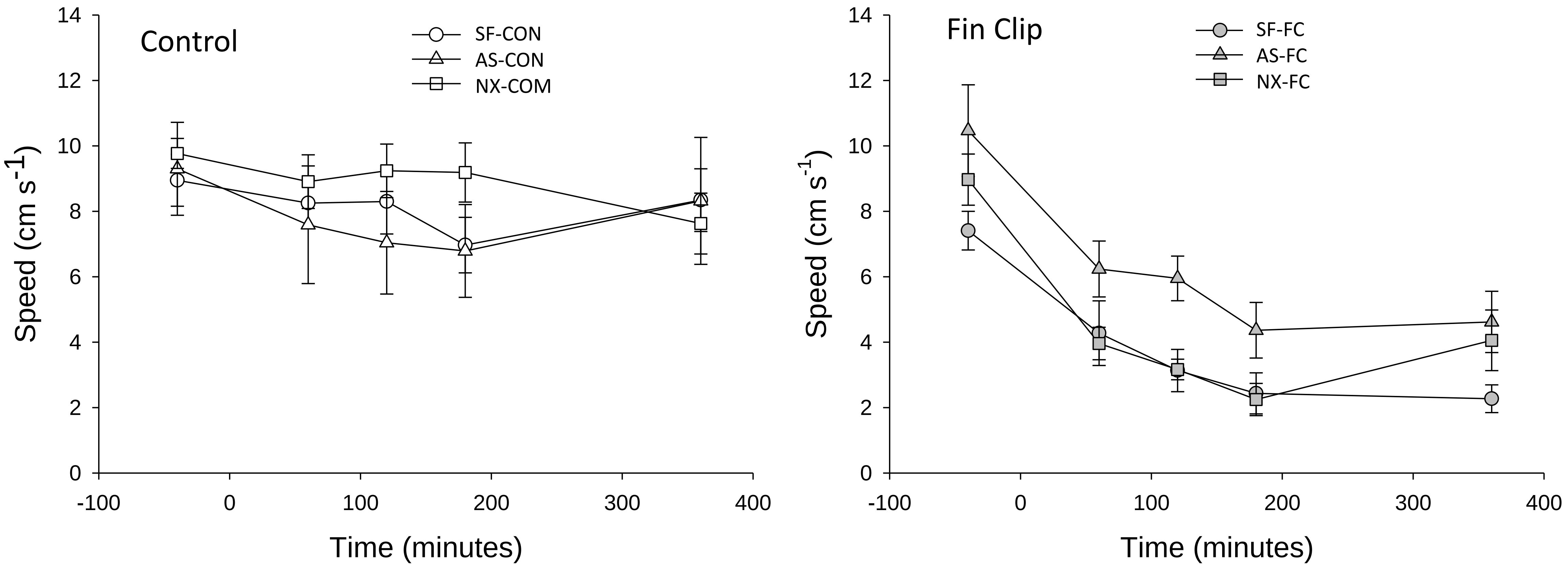

Figure S1. Mean (±SEM) speed of swimming in zebrafish subject to no fin clip as either a undisturbed Control (SF-CON), after acute stress (AS-CON) or after acute stress and administration of naloxone (NX-CON; left panel). Right panel shows same treatment groups with a fin clip (SF-FC; AS-FC, NX-FC; n = 7 per group). Observations were made prior to treatment at time 0 minutes and at subsequent intervals.
